# A new study on the growth behavior of austenite grains during heating processes

**DOI:** 10.1038/s41598-017-04371-8

**Published:** 2017-06-21

**Authors:** Dong Xu, Cheng Ji, Hongyang Zhao, Dongying Ju, Miaoyong Zhu

**Affiliations:** 1 College of Materials Science and Engineering, Hehei University of Engineering, Handan, 056038 China; 2 School of Materials and Metallurgy, University of Science and Technology Liaoning, Anshan, 114051 China; 3 Advanced Science Institute, Saitama Institute of Technology, Fukaya, 3690293 Japan; 4 School of Metallurgy, Northeastern University, Shenyang, 110819 China

## Abstract

In this paper, the effects of the heating temperature and holding time on the austenite grain growth of SCM435 steel were studied and analyzed in terms of the average, macro-axis and minor-axis size of the grains. The results indicated that the classical Sellars model was highly consistent for describing the growth stage of the new austenite but poorly described the initial nucleation-growth stage and stability stage of the austenite. A new model that expresses the average austenite grain growth of SCM435 steel was developed, and the values calculated based on this model were highly consistent with the actual measured values. The standard deviation and expected grain-size expressions increased as the heating temperature and holding time increased. The macro-axis and minor-axis size of the grains were linearly correlated with the average size, and the ratio of the macro-axis to the minor-axis size remained generally constant during grain growth. Furthermore, this paper provides a new way of thinking about heredity in materials science from the perspective of mathematical characteristics.

## Introduction

The grain size has enormous effects on the properties of metals and is determined by grain growth and grain refinement^1–5^. The austenite grain size of steel is refined during recrystallization by forging and rolling processes at high temperatures^6–11^. The grain growth primarily occurs during the heating process of the steel. Some scholars have previously performed statistical analyses of austenite grain sizes during the heating process of steel and obtained different grain growth models for different types of steel using regression^12–27^. For example, Heinze C^24^. studied the critical factors for controlling weld microstructures, and a model for austenite grain growth was developed using Sysweld software. Pous-Romero H^25^. studied the austenite grain growth in nuclear pressure-vessel steel. Zhao Y.L.^26^ Studied the kinetics of austenite grain growth in medium-carbon niobium-bearing steel, and curves of the calculated austenite average grain sizes and measured sizes for different heating temperatures were obtained.

At present, most empirical models have been based on the Sellars model^12^. However, few studies have been conducted to analyze the effects of the heating temperature and holding time on the average size, macro-axis size and minor-axis size of individual austenite grains in detail and seriatim.

SCM435 steel was selected as the research focus in this paper. SCM435 steel is a typical medium-carbon alloy steel, which is required to have demanding mechanical properties when used to manufacture Grade 12.9 automobile engine bolts, which are typical of high-end cold-headed products^28–30^. The author of this paper has previously studied the dynamic, static and metadynamic recrystallization of SCM435 steel and analyzed the characteristics of the changes in the recrystallized grain size^6–8^.

In this paper, the growth behavior of the grains was studied, and the effects of the holding time and heating temperature on the grain sizes were investigated and analyzed to predict the austenite growth using the Sellars model. We developed a new model that describes the austenite grain growth of SCM435 steel and provided the coefficients of the new model. Furthermore, in this paper, we analyzed the growth behavior of individual grains and the distribution of the grain sizes. Prediction models for the average size, macro-axis size and minor-axis size of SCM435 steel grains and their distribution were established. Taken together, these results can play a guiding role in determining the growth mechanisms of austenite grains, optimizing the grain sizes and improving the properties of SCM435 steel, thereby providing a theoretical foundation for reasonable heating, rolling and heat-treatment processes.

## Experiments

The high-strength bolt steel SCM435, which is composed of C (0.37%), Mn (0.55%), Si (0.28%), P (0.013%), S (0.01%), Cr (0.92%) and Mo (0.2%), was used for the experiments. By utilizing the appropriate corrosion formula, martensite can display better austenite grain boundaries. Generally, austenite is water cooled to obtain martensite, which is then used to obtain austenite grain boundaries. To refine the original austenite grains, a new process was adopted for this experiment. Original austenite grains were obtained from the laying head at a wire rod plant after water cooling.

The samples in this paper were collected from a reel collecting station to determine the effects of the heating temperature and holding time on the austenite grain sizes of the steel. They were not plastically deformed before heating.

The temperature, holding time, stress state and original cold deformation affected the growth of the austenite grains. However, due to limited time and the limited length of this paper, only the effects of the temperature and holding time were discussed.

The first experiment was performed to observe the effects of the heating temperature on the grain growth. The samples were heated in a furnace at 800 °C, 850 °C, 900 °C, 950 °C, 1000 °C, 1050 °C, 1100 °C, 1150 °C, 1200 °C and 1250 °C for 20 min and then quickly water cooled to the room temperature. The austenite grain boundaries were retained to observe the sizes of the austenite grains, and then, the average size, macro-axis size and minor-axis size of each grain were measured.

The second experiment was performed to observe the effects of the holding time on the grain growth. The samples were heated in a furnace at 850 °C and 1050 °C for 10 min, 20 min, 30 min, 40 min, 60 min, 90 min, 120 min and 180 min, respectively, and then quickly water-cooled to room temperature. After, the individual grains were analyzed.

**Figure 1:**
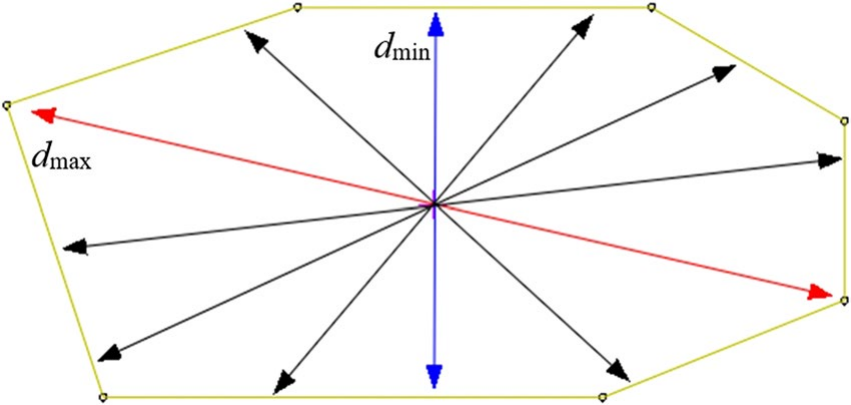
Measurement of the size of a grain.

Each metallographic image was properly processed for grain size measurements. The single average size (*d*_s_), macro-axis size (*d*_max_), and minor-axis size (*d*_min_) of the individual complete grains shown in each metallographic image were measured, as shown in Fig. 1, in which the macro-axis size (*d*_max_) was expressed in red, the minor-axis size (*d*_min_) was expressed in blue, and the average size of each grain (*d*_s_) was expressed using the arithmetic mean value of all arrows in the grain.

Different grains grow differently under the same heating conditions, and therefore the size of a single grain cannot be used for explanatory purposes. In this paper, the sizes of grains in certain areas were used to calculate the average of *d*_s_ (average size), *d*_max_ (macro-axis size), and *d*_min_ (minor-axis size) values.Here, ${\bar{d}}_{{\rm{\max }}}$ represents the arithmetic mean value of *d*_max_ of all complete grains on each metallographic image, and ${\bar{d}}_{{\rm{\min }}}$ represents the arithmetic mean value of *d*_min_ of all complete grains. In addition, ${\bar{d}}_{s}$ represents the arithmetic mean value of *d*_s_ of all complete grains, and $\bar{d}$ represents the average size of all complete grains calculated based on the total area.

The Gaussian distribution function expressed by Equation (1) was used to describe the distribution of each size.

1 $$f(x)=A\cdot \exp (-\frac{{(x-\mu )}^{2}}{2{\sigma }^{2}})$$

In this equation, *A* denotes the constant to be fitted, *μ* denotes the mathematical expectation, μm, and *σ* denotes the mathematical standard deviation (μm). Generally, ${\sigma }^{2}=\sum _{i=1}^{n}({x}_{i}-\mu )/N$.

### The construction of a new model for determining average grain sizes

#### Statistics of the average grain sizes

The grain boundaries in SCM435 steel do not corrode easily because the steel alloy contains Cr and Mo elements. The samples used for this study were analyzed using an optical microscope after corrosion using a saturated solution of picric acid mixed with a small amount of an active agent, and the metallographical images were properly processed before the grain sizes were measured. The grain sizes mentioned in this paper were calculated using the following equation:

2 $$\bar{d}=\sqrt{(\frac{S}{N})}$$

In this equation, $\bar{d}$ represents the average grain size based on the total area (μm); *S* represents the total area of all complete grains in each metallographical image (excluding the grains with an incomplete boundary along the edge of each image) (μm^2^); and *N* represents the total number of all complete grains in each metallographical image.

**Figure 2:**
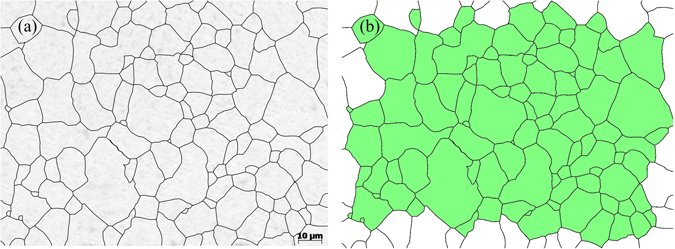
The grain size of the original austenite grain. (**a**) Metallographical image; (**b**) The area used to obtain the grain size.

Figure 2(a) shows the metallographical images of the original austenite grains obtained from the laying head after water cooling. The green area in Fig. 2(b) is the area used to calculate the average grain size. The total green area was equal to 10,627 μm^2^, and there were 95 complete grains in total. Equation (2) was used to obtain the average grain size of the original austenite: 10.58 μm (ASTM 10.5, the grain-size numbers below were all based on ASTM standards).

**Figure 3:**
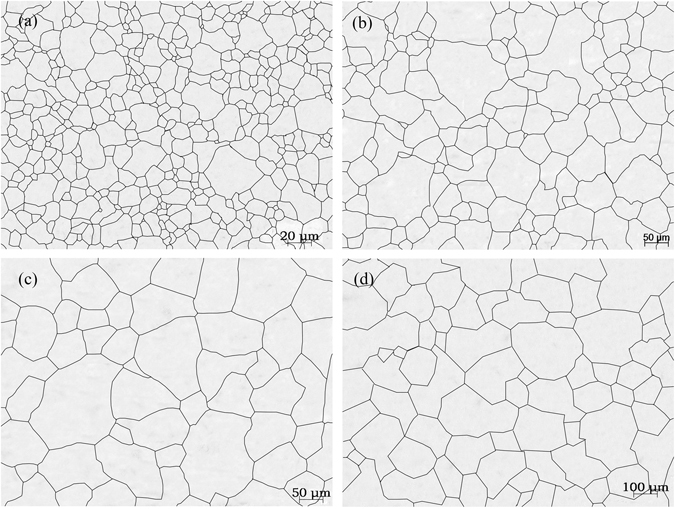
The effects of the heating temperature on the grain growth. (**a**) 800 °C; (b) 950 °C; (**c**) 1100 °C; and (**d**) 1250 °C.

Figure 3 shows the effects of the heating temperature on the austenite grain size after heating for 20 min. The experimental results indicated that the heating temperature had significant effects on the austenite grain size. From optical microscope observations, the average sizes of the austenite grains were 12.8 μm (ASTM 10) at a heating temperature of 800 °C, 30.8 μm (ASTM 7.4) at a heating temperature of 900 °C and 66.8 μm (ASTM 5.2) at a heating temperature of 1000 °C. Compared to that at 800 °C, the average size of the austenite grains at 1250 °C (133.5 μm, ASTM 3.2) was 10.4 times larger.

**Figure 4:**
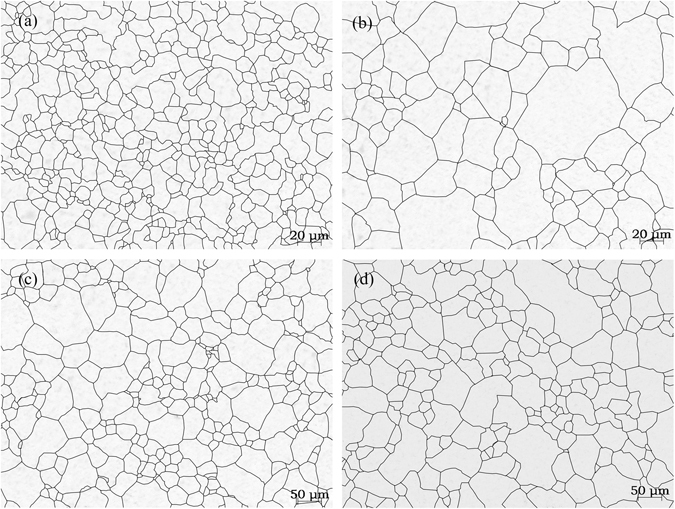
The effects of the holding time on the grain growth at 850 °C. (a) 10 min; (**b**) 40 min; (**c**) 120 min; and (**d**) 180 min.

Figure 4 shows the effects of the holding time on the grain growth at 850 °C. From the statistical analysis, $\bar{d}$ of the SCM435 steel was 11.3 μm (ASTM 10.3) after heating for 10 min and 23.4 μm (ASTM 8.2) after heating for 40 min, which was a 2.1 times increase compared to that after heating for 10 min. Moreover, $\bar{d}$ was 33.8 μm (ASTM 7.2) after heating for 120 min, and it increased to 39.2 μm (ASTM 6.7) after heating for 180 min.

**Figure 5:**
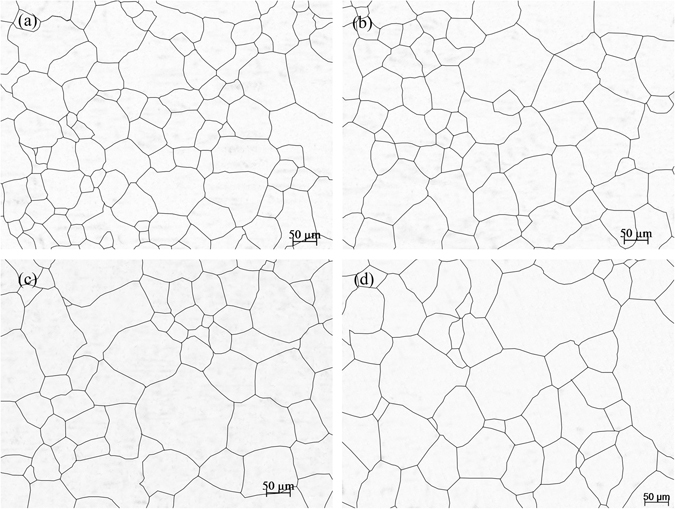
The effects of the holding time on the grain growth at 1050 °C. (**a**) 10 min; (**b**) 40 min; (**c**) 120 min; and (**d**) 180 min.

Figure 5 shows the effects of the holding time on the grain growth at 1050 °C. The statistical analysis found that $\bar{d}$ of the SCM435 steel was 59.5 μm (ASTM 5.5) after heating for 10 min, which was 5.6 times larger than the original grain size. Moreover, $\bar{d}$ was 70.5 μm (ASTM 5) after heating for 40 min, 75.5 μm (ASTM 4.8) after heating for 120 min, and 80.3 μm (ASTM 4.7) after heating for 180 min. The increase in the holding time had less of an effect on the average grain size at 1050 °C than at 850 °C.

The austenite average grain size was calculated according to Equation (2) using different heating temperatures and holding times, as shown in Table 1. As shown, the average grain size generally increased as the heating temperature and holding time increased. At 850 °C, $\bar{d}$ increased by 20 μm (2.8 times) as the holding time increased from 10 min to 90 min, and $\bar{d}$ increased by only 7.9 μm, (1.25 times) as the holding time increased from 90 min to 180 min. Therefore, there was a critical holding time for the grain growth at 850 °C of approximately 90 min, after which the grain growth decreased significantly.

**Table 1:** The statistics of $\bar{d}$ under different heating temperatures and different holding times.

Grain size, μm	Different heating temperatures, °C									
	800	850	900	950	1000	1050	1100	1150	1200	1250
20 min	11.3	14.2	29.6	51.2	58.6	63.8	85.2	103.1	112	140.4
**Grain size**, **μm**	**Different holding times**, **min**									
	10		20	30	40	60	90	120	180	
850 °C	11.3		14.2	19.4	23.4	27.1	31.3	33.8	39.2	
1050 °C	59.5		63.8	69.8	70.5	71.2	74.8	75.5	80.3	

As shown in Table 1, at 1050 °C, $\bar{d}$ increased by 10.3 μm (1.2 times) as the holding time increased from 10 min to 30 min, and $\bar{d}$ increased by only 10.5 μm (1.2 times) as the holding time increased from 30 min to 180 min. Therefore, there was a critical holding time for grain growth at 1050 °C of approximately 30 min. The grains grew more slowly after the critical holding time at 1050 °C than at 850 °C.

As shown above, the grain sizes obtained after heating at 850 °C for 10 min and at 800 °C for 20 min (11.3 μm) reached the same level as the original grain size, and the average grain size obtained after heating at 850 °C for 60 min was 2.56 times larger than the original grain size. Therefore, when measuring original grain sizes according to the Chinese standards (GB/T 6394), instead of measuring the original austenite grain size from a laying head at a wire rod plant after water cooling, an appropriate heating temperature and holding time should be selected for the various types of steel used in order to avoid large measurement errors.

#### The Sellars model for austenite grain growth

Grain growth is a spontaneous process that occurs under a certain heating temperature and holding time. Austenite grains will grow constantly as the heating temperature and holding time increase. Currently, the Sellars model has been widely used to predict the average grain sizes of austenite during the growing process^12–27^: The average grain diameter ($\bar{d}$) can be calculated according to Equation (3) for a specific heating temperature (*T*) and holding time (*t*):

3 $${\bar{d}}^{n}-{\bar{d}}_{0}^{n}=A\cdot t\cdot \exp (\frac{-Q}{RT})$$

In this equation, $\bar{d}$ represents the average grain size (μm); ${\bar{d}}_{0}$ represents the original austenite grain size (μm); *t* represents the holding time (min); *T* represents the heating temperature (K); *R* is the gas constant, which is equal to 8.31 J/(mol·K); *Q* represents the activation energy for grain growth (J/mol); and *A* and *n* are constants.

The grain growth processes for different metals have different constants, such as *A*, *n*, and *Q*. The coefficients obtained from regression using different regression software can exhibit different values. In this paper, only one type of software, 1stOpt, was used for regression.

**Figure 6:**
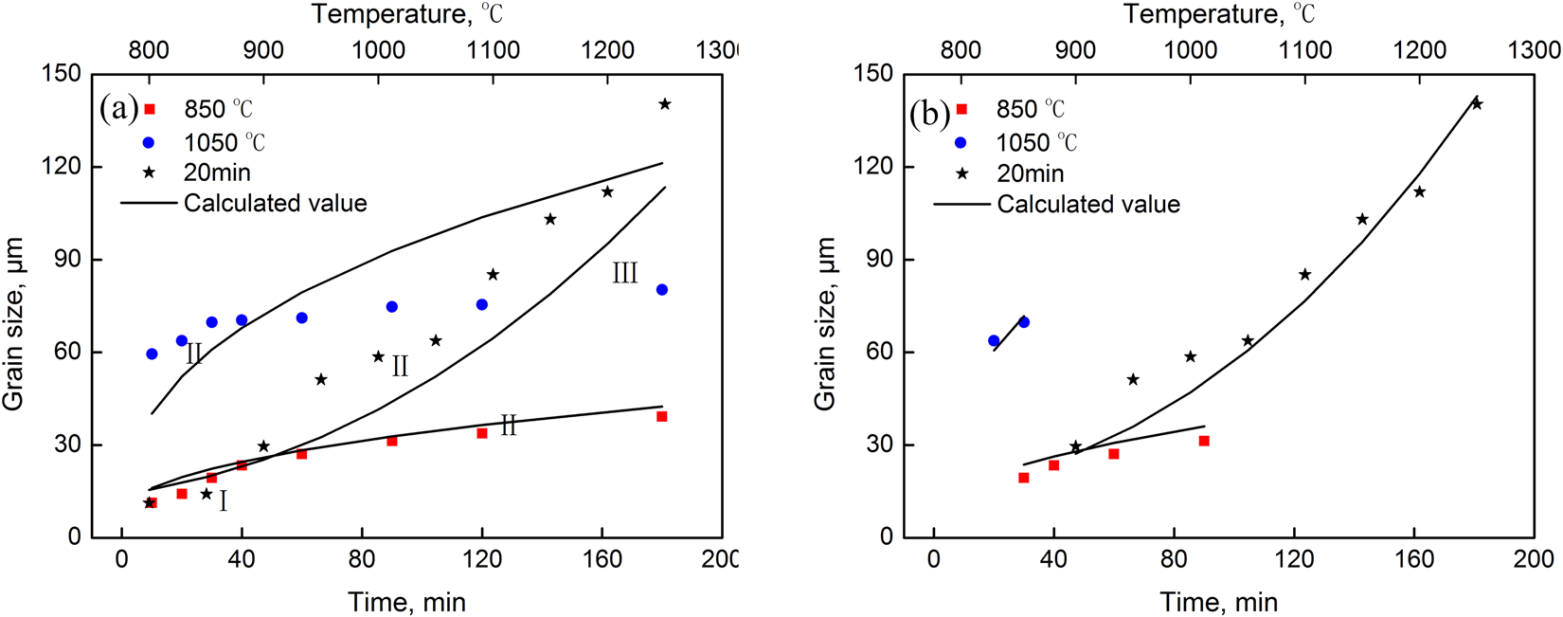
Comparison of the average grain sizes between the measured values and calculated values of the Sellars model. (**a**) Regression analysis of all data; (**b**) Regression analysis of the data from stage II.

Multiple-parameter and multiple-variable nonlinear regression fitting of Equation (3) was performed to determine the optimal values of *n* = 2.588, *A* = 6.912 × 10^9^ and *Q* = 169.4 kJ/mol. The measured values and calculated values are shown in Fig. 6(a), and their coefficient of correlation (*R*) was 0.88.

During the heating process, the growth of the austenite grains generally experiences three stages: in stage I, the austenite grains nucleate, and the nuclei grow; in stage II, new grains grow; and in stage III, the sizes of the new grains stabilize.

Owing to the logical defects, Equation (3) cannot be used to make an ideal prediction if the following three circumstances occur: (1) The new austenite grain size is not significantly greater than the original grain size; (2) the holding time is very short; and (3) the grains grow extremely slowly after being heated for a long period of time. As shown in Fig. 6(a), when used to predict the three growth stages of austenite, the Sellars model exhibited large errors.

After excluding the above three circumstances, the data from stage II were used for fitting (heating at 1050 °C for 20–30 min, at 850 °C for 30–90 min, and at 900 °C–1250 °C for 20 min) to determine the optimal values of *n* = 2.365, *A* = 4.65 × 10^9^ and *Q* = 171.2 kJ/mol. The results are shown in Fig. 6(b), with *R* equal to 0.98.

The following comparison analysis was performed between the measured grain sizes and calculated grain sizes based on the Sellars model in Fig. 6:

Stage I: the stage in which the new austenite grains nucleated and started to grow. In this stage, new austenite grains attached to the original austenite grain boundaries, inclusions, precipitates or dislocation lines nucleated quickly, and thus, a substantial number of austenite grain nuclei were formed. Additionally, because the new austenite grains were small and their grain boundaries were not likely to be impeded by the original grain boundaries, precipitates or new grain boundaries during their movement, the grains grew very quickly. In addition, the grains grew more quickly as the temperature increased. Therefore, the process of stage I became quicker as the heating temperature increased.

Because the holding time was short during this stage, the grain sizes were small at the low temperatures. Because the Sellars model is based on the original grain size, the grain sizes reached the original grain size after heating for 0 min (new austenite grains did not nucleate at this point), and increased gradually with time. Therefore, when new austenite grains did not grow sufficiently large, the Sellars model exhibited big errors, and the measured values for this stage and the values calculated based on the Sellars model were not consistent.

Stage II: the stage in which the new grains grew. Since many nuclei formed at the original austenite grain boundaries, precipitates, inclusions or dislocation lines and grew into new austenite grains, the number of nuclei became greatly reduced in this stage. Furthermore, the new austenite grains, which grew gradually, encountered the original grain boundaries, precipitates, inclusions and new austenite boundaries and grew by annexing the adjacent grains, driven by the temperature. It takes a shorter period of time to enter this stage at a higher temperature. However, this stage lasts for a shorter period of time.

As shown in Fig. 6(b), the grains gradually grew while being heated for 30~90 min at 850 °C (marked in red), for 20~30 min at 1050 °C (marked in blue) and for 20 min at 900 °C~1250 °C. The measured values for this stage were highly consistent with the values calculated according to the Sellars model.

Stage III: the stage in which the sizes of the new grains stabilized. The new austenite grains grew big that it became difficult for the temperature to drive the continual grain growth, and the grain sizes changed insignificantly with time.

As shown in Fig. 6, the new austenite grain sizes increased very slowly after being heated at 1050 °C (marked in blue) for 40 min. Because the grain sizes in the Sellars model were based on $A\cdot t\cdot \exp (\frac{-Q}{RT})$, ${\bar{d}}^{n}$ increased linearly with time at the same temperature. The measured values for this stage did not correlate well with the values calculated based on the Sellars model.

#### Construction of a new model for the austenite grain growth

Due to the limitations of the Sellars model when used to describe the growing process of the austenite grains, this paper proposed a new model, as shown in Equation (4), to describe the growth of austenite grains:

4 $$\bar{d}=f({\bar{d}}_{0})\cdot [f(t)+f(T)]$$

In this equation, $f({\bar{d}}_{0})=A{\bar{d}}_{0}$. This term represents the overall effects of the original sample matrix on the holding time and heating temperature, where ${\bar{d}}_{0}$ represents the original austenite average grain size (μm) and *A* represents the overall effects of the original austenite grain boundaries, precipitates, and inclusions and the evolving process of the dislocation behavior in the sample matrix.

The function $f(t)=\,\mathrm{ln}[B\cdot {(t+1)}^{m}]$ represents the term that describes the effects on the holding time. The grains grow more slowly with time at a certain temperature. Therefore, this term can inhibit the grain growth in a mathematical sense. In this term, *t* represents the holding time (min) and *B* and *m* represent the factors that describe the effects of the holding time on the austenite grain growth because the inhabitation of the holding time against the growing rate of the new austenite grains is first small and then big: 0 < *m* ≤ 1, in general.

As defined in the Sellars model, $f(T)={[\exp (-\frac{Q}{RT})]}^{n}$ represents the term that describes the effects of the heating temperature. Without considering the holding time (for example, the holding time is very short), the original grains grow more quickly as the heating temperature increases. Therefore, this term can promote grain growth in a mathematical sense. In this function, *T* represents the holding temperature (K), *R* is the gas constant, which is equal to 8.31 J/(mol·K), and *Q* represents the activation energy for grain growth (J/mol).

For the SCM435 steel, the above equations could be substituted in Equation (4) to obtain Equation (5):

5 $$\bar{d}=A{\bar{d}}_{0}\cdot \{\mathrm{ln}[B{(t+1)}^{m}]+{[\exp (-\frac{Q}{RT})]}^{n}\}$$

**Figure 7:**
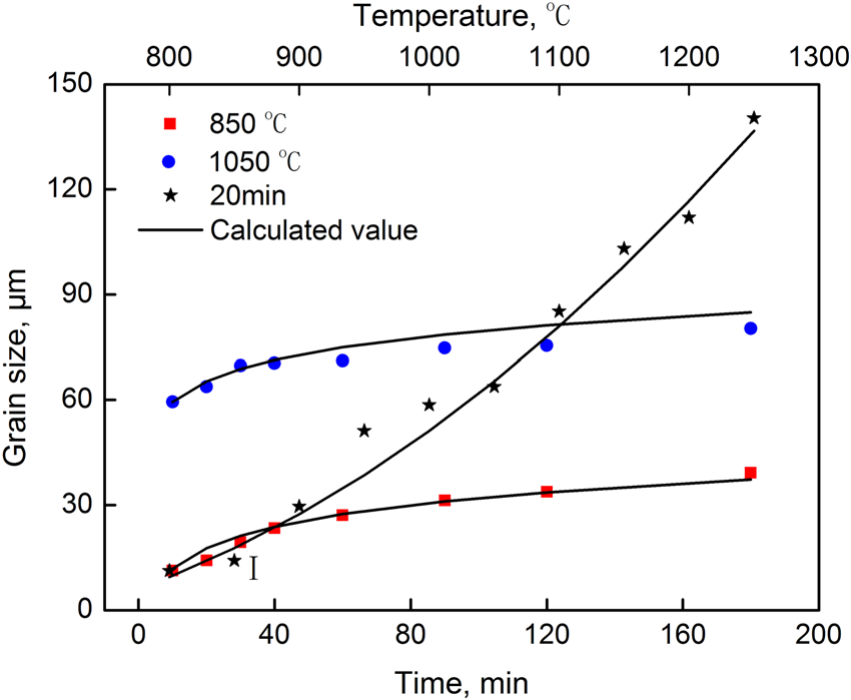
Comparison of the average grain size between the measured values and calculated values of the new model.

Multiple-parameter and multiple-variable nonlinear regression of Equation (5) was performed to determine the optimal values of *A* = 859.58, *B* = 0.9949, *m* = 0.001, *Q* = 357.6 kJ/mol, and *n* = 0.144. The measured grain size values and values calculated based on the new model are shown in Fig. 7. The *R* for these values was 0.99, which indicated that the new model was more effective than the Sellars model at predicting the growth behavior of the austenite grains of the SCM435 steel while being heated.

#### Engineering applications

Connecting rod bolts and cylinder head bolts for high-end engines require that the average grain size be smaller than 31.8 μm after heat treatment (above the grain-size number of GB 7 or ASTM 7.4). Therefore, a proper heating temperature and holding time should be selected for the heat treatment processes. Parts manufacturers often use a process that features heating at 860 °C for 60 min followed by oil quenching and tempering. The austenite grain size of the SCM435 steel used for this study was 29.2 μm according to Equation (5), which meets the requirement. Because high-end bolts are required to be 100% compliant, it is essential to add a small amount of trace elements (such as Nb and V) to steel or properly increase the Al content in order to control the growth of the austenite average grains during the heating process.

**Figure 8:**
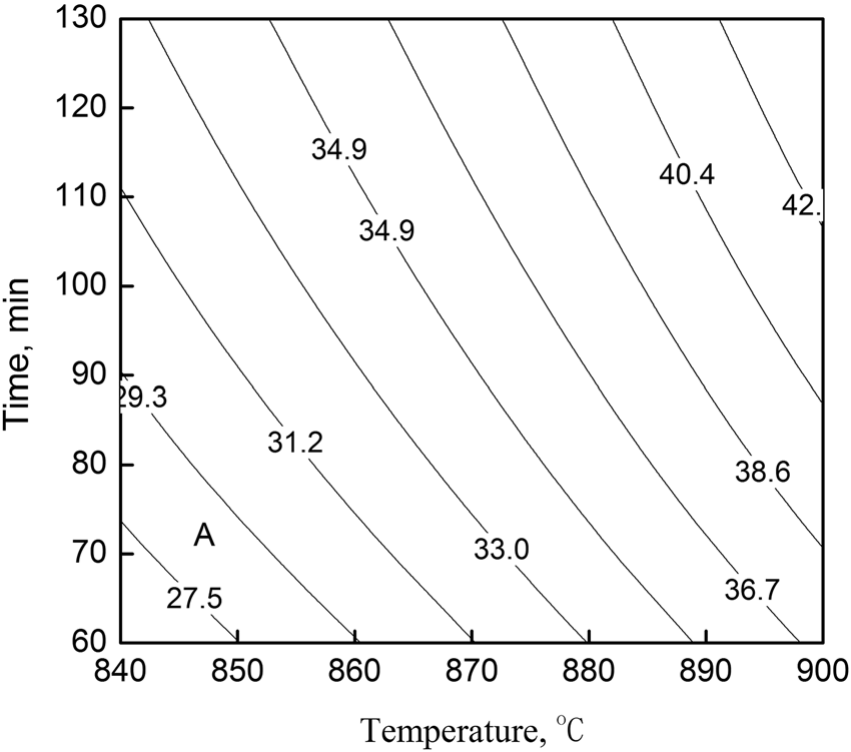
Distribution of the average grain sizes during the heating process (μm).

A process that involves heating at 840–900 °C for 60–120 min followed by oil quenching and tempering is often used to produce common bolts that contain Cr or Cr and Mo. The distribution of the average grain sizes during the heating process was obtained according to Equation (5), as shown in Fig. 8, from which it is evident that if the steel was heated at 880 °C for 60 min, the austenite average grain size would be equal to 33.1 μm. This figure can be used for predicting a suitable heat treatment process. When the average grain size is required to be smaller than 31.8 μm, a process that involves a heating zone of 870 °C or below for less than 110 min will be suitable for the steel, namely, area A shown in the figure.

### Distribution characteristics of the grain sizes

#### Statistics on the distribution of the average size of a single grain

**Figure 9:**
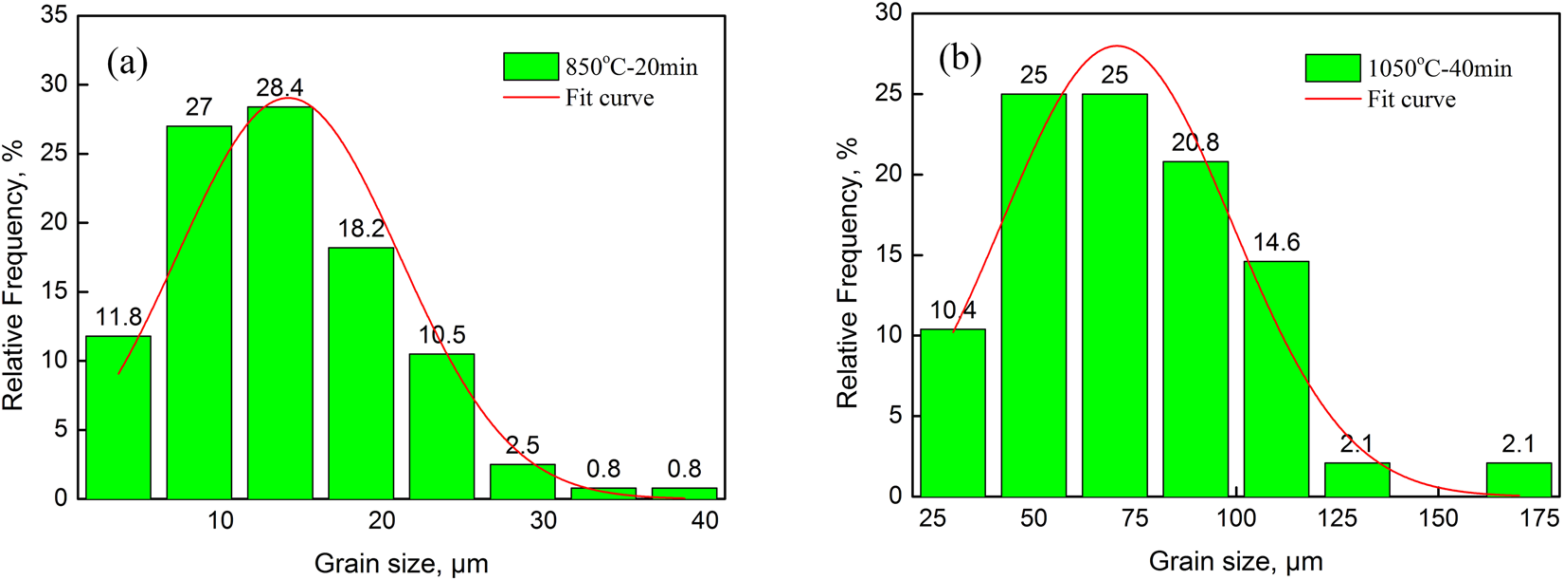
The effects of the heating conditions on the average grain size (*d*_s_). (**a**) 850 °C-20 min; (**b**) 1050 °C-40 min.

The experimental results indicated that the heating temperature and holding time had enormous effects on the single average size (*d*_s_). Figure 9(a) shows the distribution of *d*_s_ after heating at 850 °C for 20 min. Measurements of the average size of each grain indicated that the minimum *d*_s_ was 1.4 μm (ASTM 16.4), the maximum *d*_s_ was 41.1 μm (ASTM 6.6), and the arithmetic mean value (${\bar{d}}_{s}$) of all *d*_s_ values was 13.9 μm, which deviated slightly from the ${\bar{d}}_{s}$ (14.2 μm) calculated based on the total area. As illustrated in Fig. 9(a), a *d*_s_ of approximately 13.7 μm was most likely to occur, accounting for 28.4% of the relative frequency.

Since the predictions for ${\bar{d}}_{s}$ were obtained using Equation (5) and the differences between ${\bar{d}}_{s}$ and $\bar{d}$ were generally small, *μ* was expressed by $\bar{d}$ in this paper. After analyzing the distribution of *d*_s_, the standard deviation (*σ*) was calculated to be 6.88 μm. Equation (1) was regressed to obtain the value of *A* as 29.04. The regression curve is expressed by the red curve in Fig. 9(a).

Figure 9(b) shows the distribution of *d*_s_ after heating at 1050 °C for 40 min. Measurements of the average size of each grain determined that the minimum *d*_s_ was 24.6 μm (ASTM 8.1), the maximum *d*_s_ was 165.5 μm (ASTM 2.6), and the arithmetic mean value (${\bar{d}}_{s}$) of all *d*_s_ values was 72.4 μm, which deviated slightly from the ${\bar{d}}_{s}$(70.5 μm) calculated based on the total area. As illustrated in Fig. 9(b), the *d*_s_ values of approximately 50 μm and 70 μm were most likely to occur, both accounting for 25% of the relative frequency. Moreover, *μ* was expressed by $\bar{d}$ in this paper. After analyzing the distribution of *d*_s_, the standard deviation (*σ*) was calculated to be 28.51 μm. Equation (1) was regressed to obtain the value of *A* as 28. The regression curve is expressed by the red curve in Fig. 9(b).

Since the normal distribution curve fitted based on the distribution of the grain sizes simply represented the probability of the occurrence of each grain size under the heating conditions and not every predicted size corresponded to an actual grain size, the area surrounded by the curve and the x-axis in Fig. 9 was greater than 100%.

The effects of the heating conditions on the normal distribution of *d*_s_ are shown in Table 2. As shown, *A* exhibited no striking characteristics, and generally ranged between 21 and 42 with an arithmetic mean value ($\bar{A}$) of 30 μm, and *σ* generally increased as the heating temperature and holding time increased. A regression analysis of *σ* was performed using Equation (6).

6 $$\sigma =B\cdot {T}^{n}\cdot {t}^{m}$$

**Table 2:** The effects of the heating conditions on the distributions of *d*_s_, *d*_max_ and *d*_min_.

Heating temperature, °C	Holding time, min	*μ*($\bar{d}$), μm	*σ*, μm	*A*	*μ*(${\bar{d}}_{{\rm{\max }}}$), μm	*σ*_max_, μm	*A*_max_, μm	*μ*(${\bar{d}}_{{\rm{\min }}}$), μm	*σ*_min_, μm	*A*_min_
800	20	11.3	5.9	40.4	14	7.7	26.6	8.4	4.9	41.9
850	20	14.2	6.9	29	18.2	9.2	25.3	10.6	5.6	28.6
900	20	29.6	14.9	29.2	37.4	18.4	24.1	22.7	12.3	27.2
950	20	51.2	27.7	29.7	65.2	35.2	23.7	37.9	22	31.5
1000	20	58.6	33.3	36.7	70.6	45.1	36.2	43.5	26.2	18.6
1050	20	63.8	30.1	28.2	81.3	39.2	21.9	50	25	32.1
1100	20	85.2	33.9	24.7	113.6	43.8	18.1	68.6	28.3	24.3
1150	20	103.1	47.2	24.7	135.9	68.3	24.6	77.9	38.2	23.9
1200	20	112	51.2	31.4	149.2	74.4	21.5	82.6	41	28.7
1250	20	140.4	55.7	29.1	188.1	77.4	22.8	103.5	44	26
850	10	11.3	5.5	28.4	15.1	8.7	31.1	7.8	4.2	28.5
850	20	14.2	6.9	29	18.2	9.2	25.3	10.6	5.6	28.6
850	30	19.4	10.1	42.5	24.5	13.5	39.9	14.5	7.7	35
850	40	23.4	13.7	31.6	28.8	18.4	28.9	16.8	10.8	35.9
850	60	27.1	15.2	32.5	33.2	20.4	31.4	19.1	12.3	30.1
850	90	31.3	18.3	40.1	37.6	24.3	33.8	21.8	14.7	40.1
850	120	33.8	19.8	33.8	40.7	26.3	25.7	24.3	16.1	30
850	180	39.2	19.4	30.5	50.4	27	29.9	29	14.8	31.9
1050	10	59.5	27.3	23.8	75.1	33.7	23.1	47.2	22.7	26.5
1050	20	63.8	30.1	28.2	81.3	39.2	21.9	50	25	32.1
1050	30	69.8	35.1	23.2	88.1	44.2	18.4	53.9	28.8	25.7
1050	40	70.5	28.5	28	91	38	20.3	58.1	21.2	26.5
1050	60	71.2	30	28	92.2	36.5	23.1	58.5	25.4	27.1
1050	90	74.8	40.3	21.1	96	55.9	22.1	53.7	31.2	19.5
1050	120	75.5	37.5	26.4	94.7	47.5	16.9	60.2	31.3	26.8
1050	180	80.3	42.5	24	100.1	52.3	24.4	62.7	35.3	23.8

In this equation, *T* denotes the temperature (K); *t* denotes the time (min); *B*, *n* and *m* are coefficients that were calculated as 9.755 × 10^−12^, 3.954 and 0.117, respectively, from the fitting; and the coefficient of correlation (*R*) between the predicted values and actual values is equal to 0.97.

The expression of the distribution probability of the single average size (*d*_s_) was obtained from Equations (1) and (6), as expressed by Equation (7):

7 $$f({d}_{s}=30\cdot \exp (-\frac{5.25\times {10}^{21}\cdot {({d}_{s}-\bar{d})}^{2}}{{T}^{7.908}\cdot {t}^{0.234}})$$

#### Relationships of the macro-axis and minor-axis size with the average size

Alloys are difficult to corrode to obtain clear grain boundaries, as described in refs 12–27, and when data are not sufficient, it is difficult to find the limitations of the Sellars model during fitting. Therefore, few studies have been conducted that performed a systematic statistical analysis of the effects of the heating temperature and holding time on the average size, macro-axis size and minor-axis size of a single austenite grain, and few studies have been conducted to explain the correlation between the average size, macro-axis size and minor-axis size of austenite grains during their growing process.

**Figure 10:**
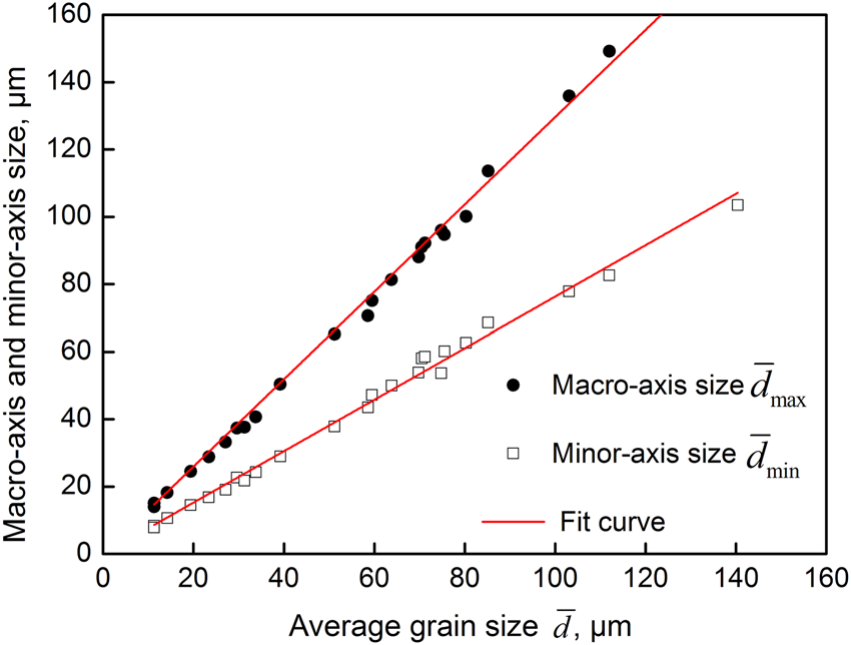
The relationships between the macro-axis and minor-axis size with the average size.

In this paper, 52 metallographic pictures were utilized (original pictures and processed pictures), and the total number of grains was approximately 3096. Then we measured and calculated the average size, macro-axis size, and minor-axis size of every grain. A statistical analysis of the macro-axis size (*d*_max_) and minor-axis size (*d*_min_) of each grain indicated that the macro-axis size (${\bar{d}}_{{\rm{\max }}}$) and minor-axis size (${\bar{d}}_{{\rm{\min }}}$) were linearly correlated with the average size ($\bar{d}$), as shown in Fig. 10. Figure 10 was regressed to the expressions of ${\bar{d}}_{{\rm{\max }}}$and ${\bar{d}}_{{\rm{\min }}}$, as described by Equations (8) and (9), respectively:

8 $${\bar{d}}_{{\rm{\max }}}=1.296\bar{d}$$

9 $${\bar{d}}_{{\rm{\min }}}=0.763\bar{d}$$

As shown in Fig. 10, the actual measured values deviated slightly from the regression curve, and the correlation coefficients between Equations (8) and (9) and the actual values were 0.998 and 0.997, respectively. In addition, the ratio of the macro-axis to the minor-axis size of the grains remained generally constant (approximately 1.7) during the growing process of the austenite grains of the SCM435 steel.

**Figure 11:**
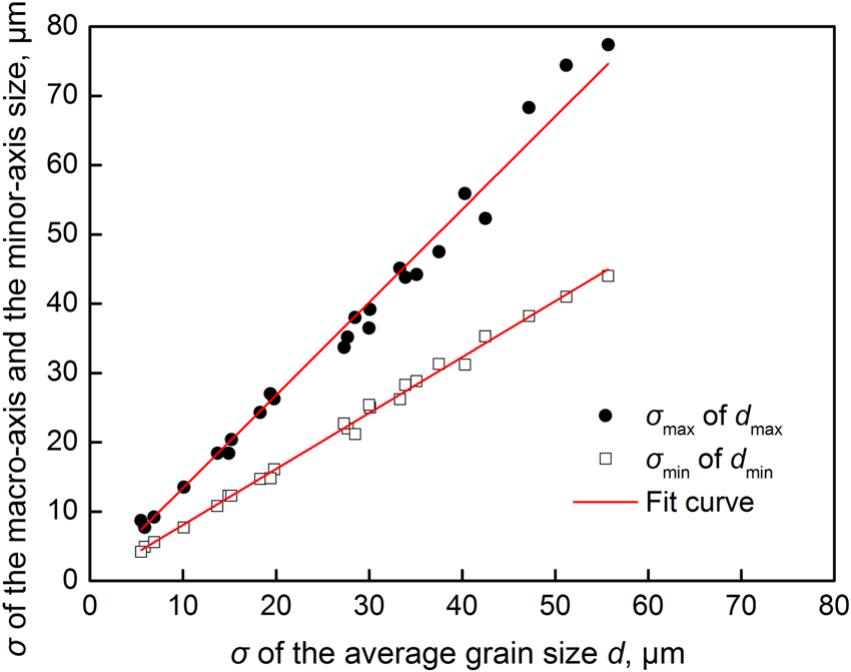
The relationships of the standard deviations of the macro-axis and minor-axis size with that of the average size.

A statistical analysis of the standard deviations of the macro-axis size (*d*_max_) and minor-axis size (*d*_min_) indicated that *σ*_max_ and *σ*_min_ were linearly correlated with the *σ* of the average size, as shown in Fig. 11. Figure 11 was regressed to the expressions of *σ*_max_ and *σ*_min_, as described by Equations (10) and (11), respectively:

10 $${\sigma }_{{\rm{\max }}}=1.34\sigma $$

11 $${\sigma }_{{\rm{\min }}}=0.807\sigma $$

#### Statistics of the distributions of *d*_max_ and *d*_min_

**Figure 12:**
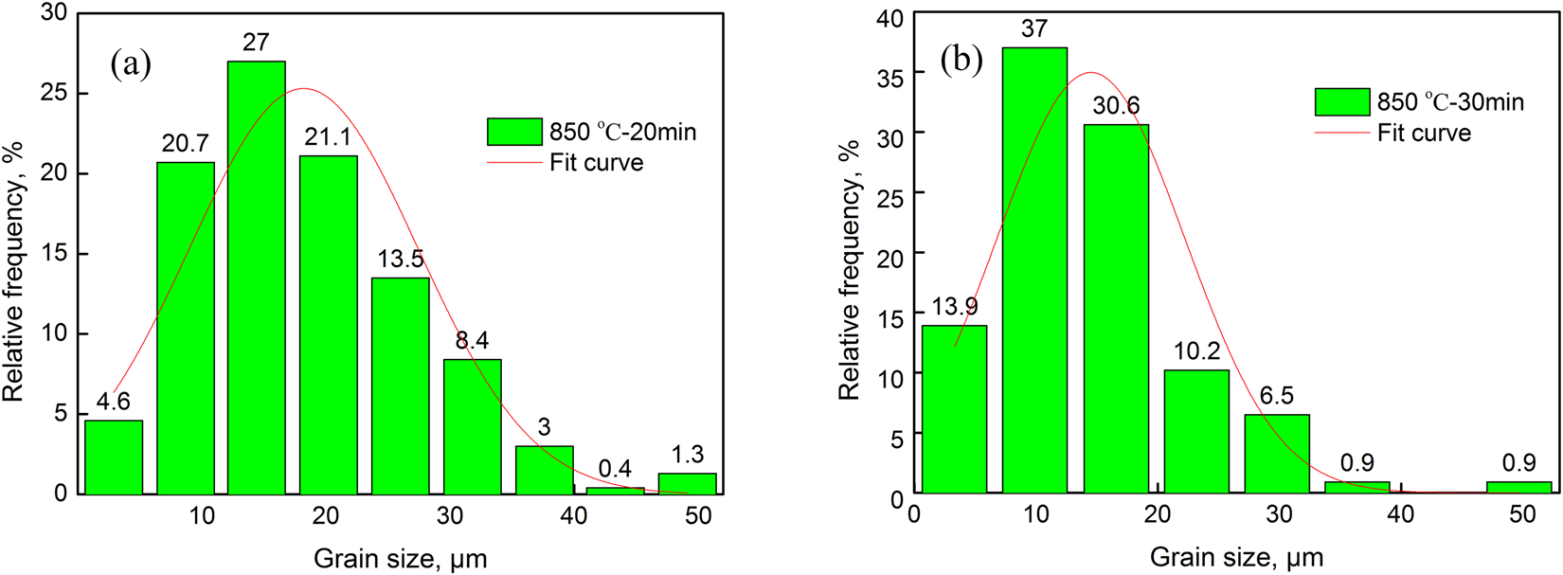
The effects of the heating conditions on the macro-axis size and minor-axis size of the grains. (**a**) Macro-axis size; (**b**) Minor-axis size.

The experimental results indicated that the heating temperature and holding time had significant effects on the macro-axis size (*d*_max_) and minor-axis size (*d*_min_) of the austenite grains. Figure 12(a) shows the distribution of *d*_max_ after heating at 850 °C for 20 min. Measurements of the *d*_max_ of each grain indicated that the minimum *d*_max_ was 2.1 μm (ASTM 15.2), the maximum *d*_max_ was 50.3 μm (ASTM 6), and the arithmetic mean value (${\bar{d}}_{{\rm{\max }}}$) of all *d*_max_ values was 18.2 μm. In this paper, *μ* was expressed by ${\bar{d}}_{{\rm{\max }}}$. As illustrated in Fig. 12(a), a *d*_max_ of approximately 14.4 μm was most likely to occur, accounting for 27% of the relative frequency. Through a regression analysis, *A*_max_ was calculated to be approximately 25.3.

Figure 12(b) shows the distribution of *d*_min_ after heating at 850 °C for 30 min. The *d*_min_ measurements of each grain indicated that the minimum *d*_min_ was 1.1 μm (ASTM 17.1), the maximum *d*_min_ was 50.7 μm (ASTM 6), and the arithmetic mean value (${\bar{d}}_{s}$) of all *d*_min_ values was 14.5 μm. As illustrated in Fig. 12(b), a *d*_min_ of approximately 10 μm was most likely to occur, accounting for 37% of the relative frequency. Through regression analysis, *A*_min_ was calculated to be approximately 25.7.

Statistical and regression analyses were performed to study the effects of the heating temperature and holding time on the distributions of *d*_max_ and *d*_min_. The *σ*, *μ* and *A* values under each condition are shown in Table 2. As shown in Table 2, σ and μ generally increased as the heating temperature and holding time increased. The relationships of *μ*_max_(${\bar{d}}_{{\rm{\max }}}$) and *μ*_min_(${\bar{d}}_{{\rm{\min }}}$) with *μ*($\bar{d}$) are shown in Equations (8) and (9), respectively. In addition, the relationships of *σ*_max_ and *σ*_min_ with *σ* are shown in Equations (10) and (11). The values of *A*_max_ and *A*_min_ demonstrated no striking characteristics. The arithmetic mean value $({\bar{A}}_{{\rm{\max }}})$ of *A*_*max*_ was 25.4 μm, and *A*_min_ generally ranged between 20 and 40, with an arithmetic mean value $({\bar{A}}_{\min })$ of 28.9.

Equations (7), (8) and (10) were combined to obtain the expression of the distribution probability of the macro-axis size (*d*_max_), as shown in Equation (12):

12 $$f({d}_{{\rm{\max }}}=25.4\cdot \exp (-\frac{2.92\times {10}^{21}\cdot {({d}_{{\rm{\max }}}-1.296\bar{d})}^{2}}{{T}^{7.908}\cdot {t}^{0.234}})$$

Equations (7), (9) and (11) were combined to obtain the expression of the distribution probability of the minor-axis size (*d*_min_), as shown in Equation (13):

13 $$f({d}_{{\rm{\min }}})=28.9\cdot \exp (-\frac{8.06\times {10}^{21}\cdot {({d}_{{\rm{\min }}}-0.763\bar{d})}^{2}}{{T}^{7.908}\cdot {t}^{0.234}})$$

#### Analysis and discussion

**Figure 13:**
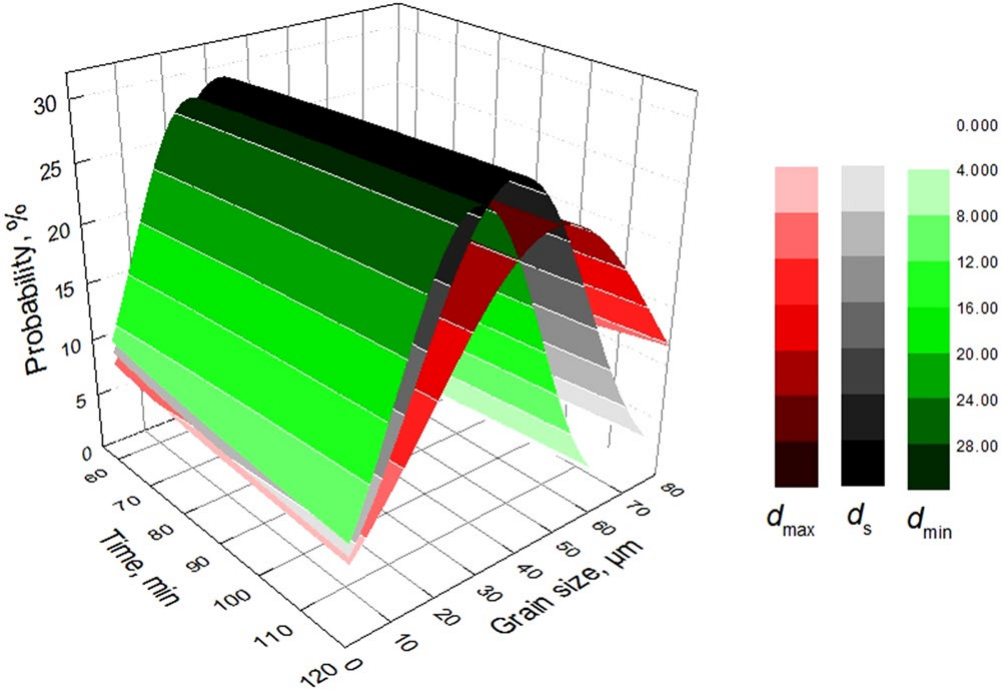
The probabilities of the occurrence of different grain sizes after heating at 860 °C.

The average size ($\bar{d}$) could be substituted in Equations (7,12 and 13) to predict the distribution of the single average size (*d*_s_), macro-axis size (*d*_max_) and minor-axis size (*d*_min_) after heating at different temperatures for different periods of time. For example, from the previous paper, the average size ($\bar{d}$) was 29.2 μm-35.5 μm after heating at 860 °C for 60 min-120 min. Figure 13 shows the probabilities of the occurrence of different grain sizes after heating at 860 °C. As shown in Fig. 13, after heating at 860 °C for 60 min, when the minor-axis size, average size and macro-axis size were 22 µm, 29 µm and 38 µm, respectively, the corresponding probability peaks were 28.9%, 30% and 25.4%, respectively; After heating at 860 °C for 120 min, when the minor-axis size, average size and macro-axis size were 27 µm, 36 µm and 46 µm, respectively, the corresponding probability peaks were 28.9%, 30% and 25.4%, respectively; The probability peaks followed the order *f* (*d*_max_) < *f* (*d*_min_) < *f* (*d*_s_) for the same holding time, and the grain sizes corresponding to the probability peaks were arranged as *d*_min_ < *d*_s <_ *d*_max_. The grain sizes corresponding to the probability peaks increased gradually as the holding time increased, but the probability peaks remained constant, primarily due to the proportional changes in the grain sizes during the heating process (Equations (8) and (9)).

As the basic unit of steel structures, the grains can greatly affect the overall properties. During heat treatment, the grain sizes and their distribution change in proportion to the original grain sizes and distribution. Therefore, the average size, macro-axis size and minor-axis size of the original grains and their distribution have significant effects of the properties of steel. This paper provides a new way of thinking about the heredity of the structures and properties of steel during processing and heat treatment from the perspective of mathematical characteristics.

## Conclusions

This paper studied the effects of the heating temperature and holding time on the average size, macro-axis size and minor-axis size of the austenite grains of SCM435 steel, and the results of the present study can be summarized as follows:

(1) The grain size obtained after heating at 850 °C for 10 min and at 800 °C for 20 min (11.3 μm) reached the level of the original grain size, and the grain size obtained after heating at 850 °C for 60 min was 2.56 times higher than the original grain size. Therefore, when measuring the original austenite grain size, an appropriate heating temperature and holding time should be selected for various types of steel in order to avoid large measurement errors.

(2) When used for predicting the austenite grain size during the heating process, the classical Sellars model has some limitations. This model produces large errors when the holding time is very short, the grain size is small or when the grains grow extremely slowly;

(3) A new expression for the average grain size of the austenite of SCM435 steel during a heating process was obtained: $\bar{d}=f({\bar{d}}_{0})\cdot [f(t)+f(T)]$. Nonlinear regression was performed to determine the optimal values of the various parameters;

(4) The ratio of the macro-axis to the minor-axis size of the grains remained generally constant during the growing process of the austenite grains (approximately 1.7).

(5) The original grain sizes and their distribution affect the properties of steel because the distribution of the grain sizes during heat treatment changes in proportion to the distribution of the original grain sizes. This paper provides a new way of thinking about the heredity of the structures and properties of steel during processing and heat treatment from the perspective of mathematical characteristics.
